# T2-FLAIR mismatch sign, an imaging biomarker for CDKN2A-intact in non-enhancing astrocytoma, IDH-mutant

**DOI:** 10.1007/s10143-024-02632-5

**Published:** 2024-08-09

**Authors:** Shumpei Onishi, Masato Kojima, Fumiyuki Yamasaki, Vishwa Jeet Amatya, Ushio Yonezawa, Akira Taguchi, Iori Ozono, Yukari Go, Yukio Takeshima, Eiso Hiyama, Nobutaka Horie

**Affiliations:** 1 Department of Neurosurgery, Graduate School of Biomedical and Health Sciences, Hiroshima University, 1-2-3 Kasumi, Minami-ku, Hiroshima City, 734-8551 Hiroshima Japan; 2 Department of Pediatric Surgery, Graduate School of Biomedical and Health Sciences, Hiroshima University, Hiroshima, Japan; 3 Natural Science Center for Basic Research and Development, Hiroshima University, Hiroshima, Japan; 4 Department of Pathology, Graduate School of Biomedical and Health Sciences, Hiroshima University, Hiroshima, Japan; 5 Medical Division Technical Center, Hiroshima University, Hiroshima, Japan

**Keywords:** T2-FLAIR mismatch sign, Glioma, Astrocytoma, IDH-mutant, CDKN2A homozygous deletion

## Abstract

**Introduction:**

The WHO classification of central nervous system tumors (5th edition) classified astrocytoma, IDH-mutant accompanied with CDKN2A/B homozygous deletion as WHO grade 4. Loss of immunohistochemical (IHC) staining for methylthioadenosine phosphorylase (MTAP) was developed as a surrogate marker for CDKN2A-HD. Identification of imaging biomarkers for CDKN2A status is of immense clinical relevance. In this study, we explored the association between radiological characteristics of non-enhancing astrocytoma, IDH-mutant to the CDKN2A/B status.

**Methods:**

Thirty-one cases of astrocytoma, IDH-mutant with MTAP results by IHC were included in this study. The status of CDKN2A was diagnosed by IHC staining for MTAP in all cases, which was further confirmed by comprehensive genomic analysis in 12 cases. The T2-FLAIR mismatch sign, cystic component, calcification, and intratumoral microbleeding were evaluated. The relationship between the radiological features and molecular pathological diagnosis was analyzed.

**Results:**

Twenty-six cases were identified as CDKN2A-intact while 5 cases were CDKN2A-HD. The presence of > 33% and > 50% T2-FLAIR mismatch was observed in 23 cases (74.2%) and 14 cases (45.2%), respectively, and was associated with CDKN2A-intact astrocytoma (*p* = 0.0001, 0.0482). None of the astrocytoma, IDH-mutant with CDKN2A-HD showed T2-FLAIR mismatch sign. Cystic component, calcification, and intratumoral microbleeding were not associated with CDKN2A status.

**Conclusion:**

In patients with non-enhancing astrocytoma, IDH-mutant, the T2-FLAIR mismatch sign is a potential imaging biomarker for the CDKN2A-intact subtype. This imaging biomarker may enable preoperative prediction of CDKN2A status among astrocytoma, IDH-mutant.

## Introduction

The WHO classification of tumors of the central nervous system was systematically updated in 2016 and 2021 based on the molecular characteristics [1, 2]. In the WHO 2016 update, astrocytoma was defined as diffuse glioma with IDH mutation and 1p/19q non-codeletion, and the WHO grade was defined based entirely on the histological features [1]. In WHO 2021, all IDH-mutant diffuse astrocytic tumors are classified into a single category “astrocytoma, IDH-mutant” and graded as 2, 3, or 4. In case of the presence of CDKN2A and CDKN2B homozygous deletion (CDKN2A/B-HD), the tumor is graded as “astrocytoma, IDH-mutant, grade 4,” even in the absence of histological features such as necrosis or microvascular proliferation [2]. This new definition is based on previous studies in which CDKN2A/B-HD was found to be associated with poor prognosis among astrocytoma, IDH-mutant [3–5]. Therefore, different treatment strategies should be established based on the status of CDKN2A/B. Currently, CDKN2A/B-HD can be examined with fluorescence in situ hybridization (FISH), multiplex ligation-dependent probe amplification (MLPA), or next-generation sequencing (NGS). However, the high cost of these investigations prevents their routine clinical use. Immunohistochemical staining for methylthioadenosine phosphorylase (MTAP) was reported as a predictive biomarker for CDKN2A in the assessment of adult-type diffuse glioma [6]. The loss of immunohistochemical staining for MTAP showed an 88% sensitivity and 98% specificity for predicting CDKN2A-HD, indicating its potential role as a good surrogate marker.

The T2-FLAIR mismatch sign was developed as a novel imaging biomarker for IDH-mutant and 1p/19q non-codeleted astrocytoma with high positive predictive value and specificity [7, 8]. Previous studies have shown that the T2-FLAIR mismatch in astrocytoma, IDH-mutant is attributable to the characteristic pathological microcystic changes in this tumor [9, 10]. Despite the high specificity, not all astrocytoma, IDH-mutant present the T2-FLAIR mismatch sign [8, 11]. Moreover, the relationship between histological grade based on WHO 2021, especially about molecular characteristics of astrocytoma, IDH-mutant, and the T2-FLAIR mismatch sign is not well-characterized in contemporary literature.

In this study, we explored the association between the T2-FLAIR mismatch sign and the CDKN2A status among non-enhancing astrocytoma, IDH-mutant with other radiological characteristics.

## Methods

### Patients

This retrospective study was approved by the institutional review board of the Hiroshima University (E2022-0038). Written informed consent was obtained from all patients. All methods were performed in accordance with the relevant guidelines and regulations. We reviewed patients with non-enhancing astrocytoma, IDH-mutant for whom IHC for MTAP was performed between January 2009 and May 2023. All patients underwent preoperative imaging study at our institute.

### Histopathological diagnosis and molecular signature analysis

Surgically resected tumor specimens were fixed in 10% phosphate-buffered formalin and embedded in paraffin blocks. Representative slides were then stained with hematoxylin-eosin reagent for standard histological diagnosis as described previously [12]. Immunohistochemical staining for all antibodies was performed using an automated immunostainer (BenchMark GX; Ventana). The primary antibodies were anti-human IDH1 R132H (1:100, Dianova, Hamburg, Germany), anti-ATRX (1: 200; Sigma-Aldrich, St. Louis, Mo., USA) and anti-MTAP mouse monoclonal antibody, clone 2G4 (1:100, Abnova). FISH analysis for 1p/19q were also performed as described previously [12].

Tumors were diagnosed based on the WHO classification for central nervous system tumors updated in 2021 by consensus of two authors (V.J.A, & Y.T.). Tumor molecular profiling including CDKN2A/B was confirmed by NGS-based CGP test using FoundationOne^®^CDx or Oncomine™ Childhood Cancer Research Assay.

### MR acquisition and evaluation

MR scans were acquired using 3.0 T scanners (Ingenia CX 3.0 T; Philips Healthcare, Best, Netherlands or Signa Excite HD 3.0 T; GE Medical Systems, Milwaukee, WI, USA). In 18 cases (14 cases of CDKN2A-intact astrocytoma and 4 cases of CDKN2A-HD), the MR scans were performed with Ingenia CX 3.0 T. In 13 cases (12 cases of CDKN2A-intact astrocytoma and one case of CDKN2A-HD), the MR scans were performed with Signa Excite HD 3.0 T. MRI scans were independently assessed by two authors (S.O and F.Y). The following MRI sequences were evaluated for all patients: T1-weighted imaging (WI), T2-WI, T2*-WI, fluid-attenuated inversion recovery (FLAIR), and post-contrast T1WI sequencing.

The details of Ingenia CX 3.0 T image acquisition are described below. Non-enhanced T1-WI (repetition time [TR]: 500 ms, echo time [TE]: 10 ms; field of view [FOV]: 220 mm, RFOV: 82.03%; matrix scan: 288, reconstruction 512; number of excitations [NEX]: 1; section thickness: 5 mm; intersection gap: 1.0 mm; and 2 acquisitions); T2-WI (TR: 3,000 ms; TE: 100 ms; echo train length: 15, FOV: 220 mm, RFOV 80%; matrix scan: 512, reconstruction 600; NEX: 2; section thickness: 5 mm; intersection gap: 1.0 mm; and 1 acquisition); T2*-weighted imaging (TR: 600 ms; TE: 16 ms; flip angle (FA) 20 deg; echo train length: 15, FOV: 220 mm, RFOV 80%; matrix size: 320, reconstruction 400; NEX: 1; section thickness: 5 mm; intersection gap: 1.0 mm; and 1 acquisition; scan time: 1 min and 19 s); and FLAIR imaging (TR: 10,000 ms; TE: 130 ms; inversion recovery time [TI]: 2600 ms; FOV: 220 mm, RFOV 78.91%; matrix size: 288, reconstruction 512; NEX: 1; section thickness: 5 mm; intersection gap: 1.0 mm; and 3 acquisitions; scan time: 3 min and 0s). Spin-echo T1-weighted images were acquired after intravenous administration of 0.1 mmol/kg of gadolinium (Gd)-based contrast agents with the following parameters: TR: 500 ms, TE: 10 ms; FOV: 220 mm, RFOV: 82.03%; matrix scan: 288, reconstruction 512; NEX: 1; section thickness: 5 mm; intersection gap: 1.0 mm; and 2 acquisitions.

The details of Signa Excite HD 3.0 T image acquisition are described below. Non-enhanced T1-WI (TR: 450 ms, TE: 18 ms; FOV: 220 × 220 mm, matrix size: 288 × 192, NEX: 1; section thickness: 6 mm; intersection gap: 1.0 mm; and 2 acquisitions); T2-WI (TR: 4,800 ms; TE: 100 ms; echo train length: 18, FOV: 220 × 220 mm; matrix scan: 512 × 320; NEX: 2; section thickness: 6 mm; intersection gap: 1.0 mm; and 1 acquisition); T2*-weighted imaging (TR: 600 ms; TE: 12 ms; FOV: 220 × 220 mm; matrix size: 320 × 192, NEX: 1; section thickness: 6 mm; intersection gap: 1.0 mm; and 1 acquisition); and FLAIR imaging (TR: 10,000 ms; TE: 140 ms; TI: 2400 ms; FOV: 220 × 220 mm; matrix size: 288 × 160; NEX: 1; section thickness: 5 mm; intersection gap: 1.0 mm; and 2 acquisitions). Spin-echo T1-weighted images were acquired after intravenous administration of 0.1 mmol/kg of Gd-based contrast agents with the following parameters: TR: 450 ms, TE: 18 ms; FOV: 220 × 220 mm; matrix scan: 288 × 192; NEX: 1; section thickness: 6 mm; intersection gap: 1.0 mm; and 2 acquisitions.

The T2-FLAIR mismatch represented (1) a complete/near-complete hyperintense signal on T2WI and (2) relatively hypointense signal on FLAIR except for a hyperintense peripheral rim [7]. The extent of tumor proportion of T2-FLAIR mismatch lesion was stratified as previously described: >33% or > 50% [13]. We also considered additional imaging features for accurate identification of the T2-FLAIR mismatch sign as previously described [14]. Necrotic cavities do not represent the T2-FLAIR mismatch sign, whereas small cysts do not sufficiently satisfy the criteria for T2-FLAIR mismatch. The T2-FLAIR mismatch sign is typically accompanied by little or no contrast enhancement. Furthermore, the degree of FLAIR signal suppression may be inhomogeneous within the tumor.

Both investigators independently assessed the T2-FLAIR mismatch sign, contrast-enhancement, cystic component, calcification, and intratumoral hemorrhage. T2-FLAIR mismatch sign was evaluated with T2WI and FLAIR image. Intratuomral microbleeding was defined as hypo-intensity on T2*WI excluding calcification assessed by CT images.

### Statistical analysis

Fisher’s exact test was used to compare the patient characteristics and to assess the association of radiological features with molecular and pathological characteristics. Inter-reviewer agreement of T2-FLAIR mismatch sign was evaluated using the Kappa statistic (κ = 0–0.40, poor; κ = 0.41–0.60, moderate; κ = 0.61–0.80, good; κ = 0.81–1.00, excellent). The diagnostic performance of each parameter for T2-FLAIR mismatch sign was evaluated by receiver operating characteristic curve analysis. Statistical analyses were performed with JMP pro ver. 15.0 (SAS institute, Cary, NC, USA). *P* values < 0.05 were considered indicative of statistical significance.

## Results

### Clinical and molecular pathological features

This study included 31 patients (median age at primary surgery: 41 years [range 19–70]) with non-enhancing astrocytoma, IDH-mutant. Based on WHO 2016 classification, the histopathological diagnoses were as follows: 22 cases of diffuse astrocytoma, IDH-mutant (grade II); 7 cases of anaplastic astrocytoma, IDH-mutant (grade III); and 2 cases of glioblastoma, IDH-mutant (grade IV). According to WHO 2021, the tumors were reclassified as follows: 20 cases of “astrocytoma, IDH-mutant, grade 2”; 4 cases of “astrocytoma, IDH-mutant, grade 3”; and 7 cases of “astrocytoma, IDH-mutant, grade 4.” Of the 7 cases of astrocytoma, IDH-mutant, grade 4, two cases were classified based on the pathological features (microvascular proliferation and necrosis) and 5 were classified based on the molecular features (CDKN2A/B HD). All cases showed immunohistochemical positivity for anti-IDH1-R132H. Twenty-seven out of the 31 cases presented loss of ATRX, while the other 4 cases showed 1p/19q non-codeletion. Based on the molecular makers, all cases were diagnosed as “astrocytoma, IDH-mutant.” Twenty-six cases of astrocytoma showed MTAP staining and were classified as CDKN2A-intact, while the other cases with loss of MTAP staining were classified as CDKN2A-HD. The status of CDKN2A/B was further confirmed with cancer genome profiling (CGP) test in 12 cases (7 cases were analyzed with FoundationOne^®^CDx and 5 cases were analyzed with Oncomine™ Childhood Cancer Research Assay). The CGP test were performed using the samples from initial surgery in 8 cases and using the recurrent samples in 4 cases. The status of CDKN2A was concordant with the status of immunohistochemical (IHC) staining for MTAP in all cases. The pathological grade of CDKN2A-HD astrocytoma based on WHO 2016 was grade II in 2 cases and grade III in 3 cases. Pathological microcystic change and pathological calcification did not show statistical difference. The ki-67 index had a tendency to be higher in astrocytoma with CDKN2A-HD, but the difference did not reach the statistical significance (*p* = 0.0737).

The summary of all cases is shown in Table 1.

**Table 1:** Summary of our case series of IDH-mutant astrocytoma

	CDKN2A-intact	CDKN2A-HD	*p*-value	
*N*	26	5		
WHO grade 2016 (GII, III, IV)	20 / 4 / 2	2 / 3 / 0		
WHO grade 2021 (G2, 3, 4)	20 / 4 / 2	0 / 0 / 5		
Age	39.96 ± 10.97	38.80 ± 16.47	0.8426	
Gender (M: F)	18 : 8	4 : 1	0.3875	
Radiological characteristics				
T2-FLAIR mismatch (> 50%)	14 (53.8%)	0 (0%)	0.0482	*
T2-FLAIR mismatch (> 33%)	23 (88.5%)	0 (0%)	0.0003	*
Cyst	5 (19.2%)	0 (0%)	0.5601	
Calcification	6 (23.1%)	0 (0%)	0.5533	
Intratumoral microbleeding	5 (19.2%)	1 (20.0%)	1.000	
Pathological characteristics				
Microcystic change	13 (50.0%)	2 (40.0%)	1.000	
Calcification	6 (23.1%)	1 (20.0%)	1.000	
ki-67 index (median (range))	5.0% (1.0–40.0%)	15.0% (2.0–75.0%)	0.0737	

### The status of CDKN2A and radiological characteristics

The presence of > 33% and > 50% T2-FLAIR mismatch was observed in 23 cases (74.2%) and 14 cases (45.2%), respectively. The inter-reviewer agreement for the T2-FLAIR mismatch sign was excellent (κ = 1.00). All cases showed no or mild contrast enhancement with gadolinium.

All cases with positive T2-FLAIR mismatch sign had CDKN2A-intact astrocytoma. The presence of > 33% and > 50% T2-FLAIR mismatch was associated with CDKN2A-intact astrocytoma (*p* = 0.0003, 0.0482). The sensitivity, specificity, and positive predictive value of > 33% T2-FLAIR mismatch sign for CDKN2A-intact astrocytoma were 88.5%, 100%, and 100% (AUC = 0.942), respectively. The sensitivity, specificity, and positive predictive value of > 50% T2-FLAIR mismatch sign for CDKN2A-intact astrocytoma were 53.8%, 100%, and 100% (AUC = 0.769), respectively. The T2-FLAIR mismatch sign was negative for all cases of astrocytoma with CDKN2A-HD.

The locations of CDKN2A-intact astrocytoma were frontal lobe in 16 cases, temporal lobe in 7 cases, insula in 2 cases, and occipital lobe in 1 case. All the CDKN2A-HD astrocytomas were located in the frontal lobe (5 cases). Radiological cystic component, calcification, and intratumoral microbleeding were not associated with CDKN2A status (*p* = 0.5601, 0.5533 and 1.000, respectively).

**Fig. 1:**
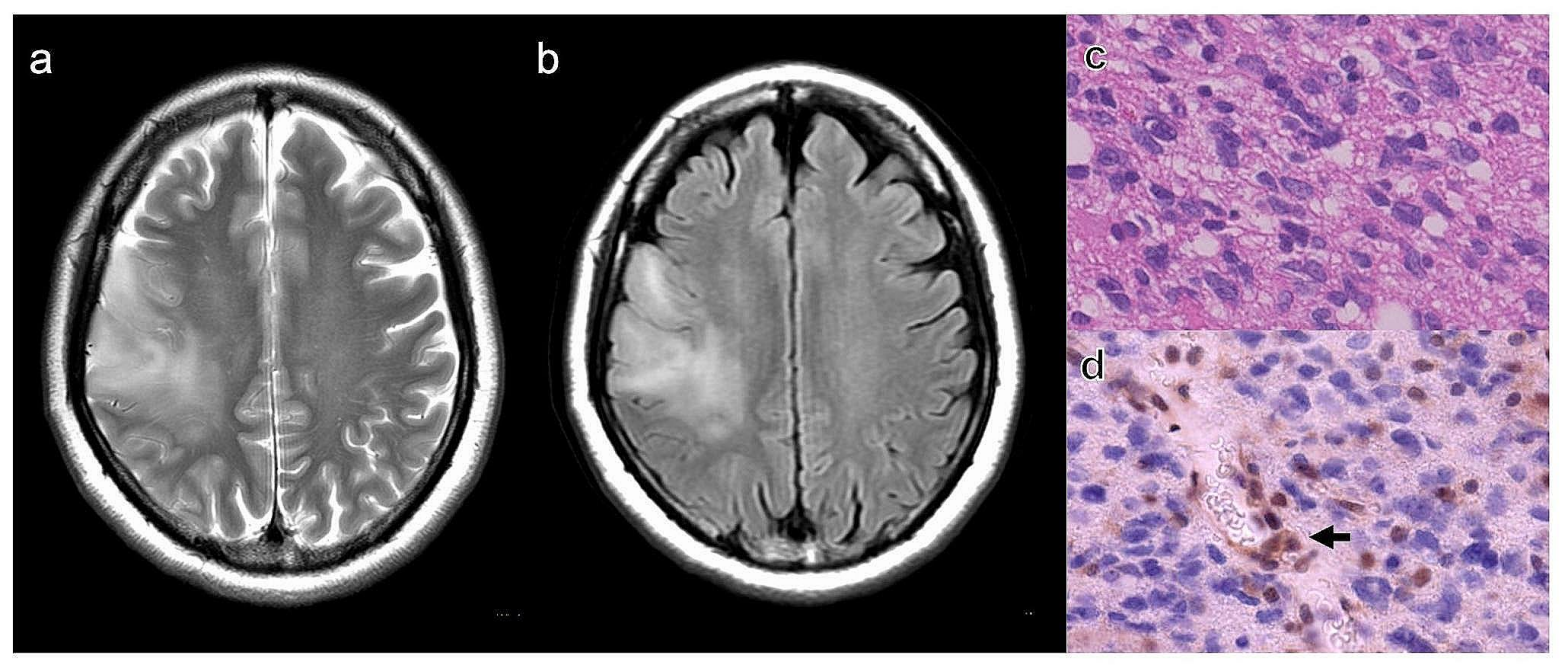
Representative case of astrocytoma, IDH-mutant with CDKN2A-HD. Axial T2WI (**A**) and FLAIR (**B**) images of patient with right frontal *IDH*-mutant astrocytoma. The tumor shows hyper-intensity signal on T2WI and FLAIR. Hematoxylin and eosin-stained section showing astrocytoma (**C**). Immunohistochemical staining shows loss of MTAP expression. Black arrow indicates vascular endothelium (internal control) (**D**)

**Fig. 2:**
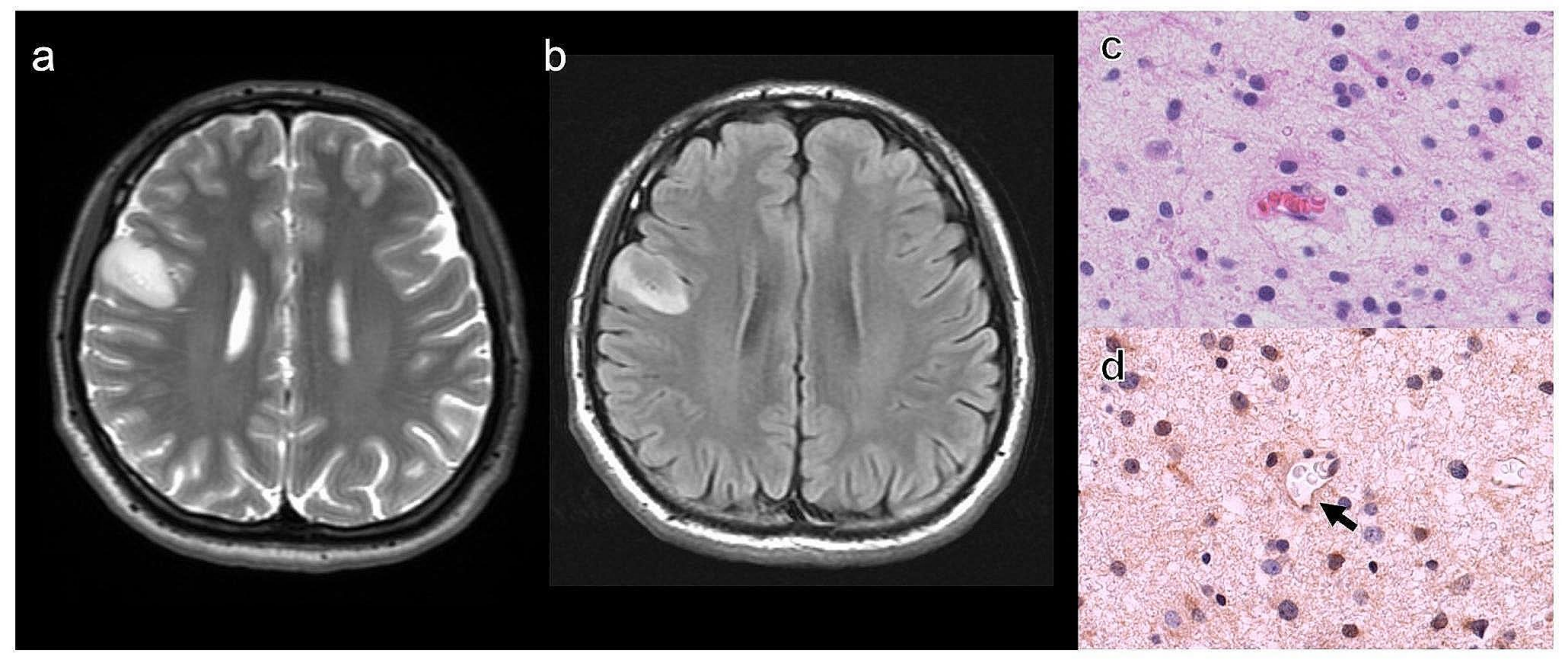
Representative case of astrocytoma, IDH-mutant with CDKN2A-intact. Axial T2WI (**A**) and FLAIR (**B**) images of patient with right frontal *IDH*-mutant astrocytoma showing the T2-FLAIR mismatch sign. T2WI demonstrates homogeneous hyperintense signal throughout the lesion (A). FLAIR displays relatively hypointense signal in majority of the lesion with peripheral hyperintense signal rim (B). Hematoxylin and eosin-stained section shows astrocytoma (**C**). Immunohistochemical staining shows retained MTAP staining. Black arrow indicates vascular endothelium (internal control) (**D**)

The relationship between radiological characteristics and CDKN2A status is also shown in Table 1. A representative case of astrocytoma, IDH-mutant with CDKN2A-HD is presented in Fig. 1. A representative case of astrocytoma, IDH-mutant with CDKN2A-intact is shown in Fig. 2.

## Discussion

In this study, we investigated the association between T2-FLAIR mismatch sign and CDKN2A status among non-enhancing astrocytoma, IDH-mutant, and found that T2-FLAIR mismatch sign was correlated with CDKN2A-intact astrocytoma.

T2-FLAIR mismatch sign was reported as the novel imaging biomarker for astrocytoma, IDH-mutant and 1p/19q non-codeletion [7], and several studies have validated the reliability of this sign [14]. Although some brain tumors (such as dysembryoplastic neuroepithelial tumor (DNET) and diffuse midline glioma [15–17]) also present the T2-FLAIR mismatch sign, this sign highly specific for astrocytoma, IDH-mutant among patients with lower grade glioma [8, 11]. In a recent meta-analysis about T2-FLAIR mismatch sign among adult patients with low-grade glioma, the pooled specificity of T2-FLAIR mismatch sign for astrocytoma, IDH-mutant was 99% (95% CI: 96–100%) [8]. In another meta-analysis including DNET, pediatric-type gliomas, and nonneoplastic lesions, the pooled specificity was 100% (95% CI, 88–100%) [11].

Several studies reported that the T2-FLAIR mismatch region in astrocytoma, IDH*-*mutant was pathologically associated with the microcystic change [9, 10]. However, the molecular implications of the mismatch sign have not yet been clarified. Furthermore, not all astrocytoma, IDH-mutant present the T2-FLAIR mismatch sign. In a recent meta-analysis, the sensitivity of T2-FLAIR mismatch sign for astrocytoma, IDH-mutant was found to be 42% (95% CI: 34–50%) among adult lower grade gliomas [8]. Therefore, determining the different molecular implications of the presence or absence of T2-FLAIR mismatch sign in astrocytoma, IDH-mutant is of much clinical relevance. In this study, we observed a correlation between presence of T2-FLAIR mismatch sign and CDKN2A-intact in non-enhancing astrocytoma, IDH-mutant.

IDH-mutant gliomas are associated with longer survival compared to IDH-wildtype gliomas [18]. However, patients with astrocytoma, IDH-mutant with CDKN2A/B-HD have shorter survival (expected median OS: approximately 3 years) which corresponds to WHO CNS grade 4 [19]. These different prognostic factors have a significant impact on treatment decision-making. Therefore, the ability to predict the status of CDKN2A can help inform individual treatment planning in clinical settings. The T2-FLAIR mismatch sign is one of the potential predictors of CDKN2A status in patients with non-enhancing astrocytoma, IDH-mutant. However, the absence of T2-FLAIR mismatch sign does not completely predict astrocytoma with CDKN2A-HD. Therefore, other imaging biomarkers are required for more accurate differentiation among diffuse gliomas.

In recent years, MTAP was reported as a useful immunohistochemical surrogate marker for CDKN2A-HD in adult-type infiltrating diffuse astrocytoma (sensitivity: 88.2%, specificity: 98.3%) [6]. A high correlation of MTAP immunohistochemical reactivity with CDKN2A homozygous deletion (as determined by FISH) was first reported in malignant mesothelioma with a sensitivity of 78% and specificity of 98% [20]. Because the MTAP gene is located at the 9p21 locus adjacent to the *CDKN2A* gene [21], immunohistochemical loss of MTAP could be a good surrogate marker of CDKN2A-HD. In this study, the T2-FLAIR mismatch sign was associated with the expression of MTAP in *IDH*-mutant astrocytoma.

Some limitations of our study should be acknowledged. This was a retrospective study involving a small number of patients with astrocytoma, IDH-mutant with CDKN2A-HD. In previous studies about CDKN2A status in IDH-mutant gliomas, 11–18% of astrocytoma, IDH-mutant showed CDKN2A-HD [3, 22, 23]. In our study, 19% of astrocytoma, IDH-mutant showed CDKN2A-HD, which was consistent with the previous studies. Moreover, CGP test was not performed in all patients to confirm the status of CDKN2A/B. Furthermore, we excluded gadolinium-enhancing astrocytoma, IDH-mutant because of the difficulty in evaluating the T2-FLAIR mismatch sign in such cases. With respect to the parameters of FLAIR image acquisition, inversion time (TI) may influence the sensitivity of T2-FLAIR mismatch sign [24]. In our study, FLAIR images were acquired with two different TI (2,400 and 2,600 ms), which may have influenced the results. Nonetheless, our data has important implications for the molecular profile of non-enhancing astrocytoma, IDH-mutant with T2-FLAIR mismatch sign. A larger prospective study is required to confirm the usefulness of T2-FLAIR mismatch sign for predicting the molecular features.

## Conclusion

Among non-enhancing astrocytoma, IDH-mutant, the T2-FLAIR-mismatch sign is a potential imaging biomarker for CDKN2A-intact subtype. This imaging biomarker may enable preoperative prediction of the CDKN2A status among patients with astrocytoma, IDH-mutant.

## Data Availability

The datasets generated during and/or analyzed during the current study are available from the corresponding author on reasonable request.
